# ToF-SIMS analysis of osteoblast-like cells and their mineralized extracellular matrix on strontium enriched bone cements

**DOI:** 10.1186/1559-4106-8-17

**Published:** 2013-07-23

**Authors:** Julia Kokesch-Himmelreich, Matthias Schumacher, Marcus Rohnke, Michael Gelinsky, Jürgen Janek

**Affiliations:** 1 Institute for Physical ChemistryJustus - Liebig - University GiessenHeinrich-Buff-Ring 5835392GiessenGermany; 2 Centre for Translational Bone, Joint and Soft Tissue ResearchTechnische Universität DresdenFetscherstrasse 7401307DresdenGermany

**Keywords:** Strontium doped bone cements, Osteoblast-like cells, Mineralized extracellular matrix, ToF-SIMS, Strontium uptake in cells, Strontium incorporation in mineralized ECM

## Abstract

Commonly used implants for therapeutic approaches of non-systemically impaired bone do not sufficiently support the healing process of osteoporotic bone. Since strontium (II) has been proven as an effective anti-osteoporotic drug new types of strontium enriched calcium phosphate bone cements were developed. As osteoporosis is characterized by an imbalance of osteoblast and osteoclast activity the influence of this newly generated strontium enriched biomaterials on the cellular behavior of osteoblast-like cells was investigated by time of flight secondary ion mass spectrometry (ToF-SIMS). ToF-SIMS is used to analyze whether strontium is incorporated in the mineralized extracellular matrix (mECM) and whether there is strontium uptake by osteogenically differentiated human mesenchymal stem cells (hMSCs). Therefore hMSCs were cultured in osteogenic differentiation medium for 21 days on two different strontium enriched bone cements (S100 and A10) and for reference also on the pure calcium phosphate cement (CPC) and on a silicon wafer. The distribution of strontium in the osteoblast-like cells and within their mineralized extracellular matrix was analyzed. A higher intensity of the strontium signal could be detected in the region of the mECM, synthesized by cells cultivated on the Sr- substituted bone cement (S100) in comparison to the reference groups. The osteoblast-like cells used the released strontium from the biomaterial to synthesize their mECM. Apart from that a uniform strontium distribution was measured within all investigated cells. However, different amounts of strontium were found in cells cultured on different biomaterials and substrates. Compared to the negative controls the strontium content in the cells on the strontium enriched biomaterials was much higher. A higher concentration of strontium inside the cells means that more strontium can take part in signaling pathways. As strontium is known for its beneficial effects on osteoblasts by promoting osteoblastic cell replication and differentiation, and reducing apoptosis, the newly developed strontium enriched calcium phosphate cements are promising implant materials for osteoporotic bone.

**Electronic supplementary material:**

The online version of this article (doi:10.1186/1559-4106-8-17) contains supplementary material, which is available to authorized users.

## Background

Osteoporosis is the most common type of systemic bone disease. It is characterized by reduction of bone mineral density (BMD) and leads to an increased risk of fracture [1]. Commonly used implants for therapeutic approaches of non-systemically impaired bone are not sufficient in supporting the healing process of damaged osteoporotic bone regarding their chemical, biological and biomechanical properties [2]. Therefore implants and biomaterials which are appropriate for the therapeutic use in systemically altered bone are currently under development. Calcium phosphate cements, which have been proposed to reinforce osteoporotic bone, are particularly promising [3]. In general bone cements have been used very successfully in the treatment of a wide range of bone defects due to their biological beneficial behavior and handling properties [4]. They perfectly fit into the bone defect cavity or can be molded easily to the desired shape [5]. It was proven that calcium phosphate materials also allow ionic substitution, which offers another degree of freedom in the development of improved cements. Ions with specific activity and with biological relevance, such as Mg^2+^ and Zn^2+^ can be added easily to the precursor materials [6]. Adding an agent with anti-osteoporotic effects could improve the osseointegration of the implant which would cause a better fracture healing [7]. Strontium ions have been proven as an effective anti-osteoporotic drug through their antiresorptive and bone-forming effects [8]. *In vitro* experiments showed that strontium enhances the proliferation of preosteoblastic cells and bone matrix synthesis [9]. And it was also found that it reduces the osteoclast activity [10]. Furthermore it has been demonstrated that strontium is incorporated into mineralized nodules without any negative effect on the formation of mineralized matrix even in long-term treatment of osteoblast like cells with strontium ranelate in the growth medium [11]. It has also been demonstrated that strontium stimulates bone formation and decreases bone resorption *in vivo*[12, 13]. Clinical studies in postmenopausal osteoporotic patients showed a beneficial effect of orally administered strontium ranelate on fracture risk and micro architecture [14, 15]. Therefore strontium ranelate is increasingly used in treatment strategies for osteoporosis [16]. Recently Li et al. found an improvement of implant osseointegration in osteoporotic rats, which had an additional oral dispense of strontium ranelate [17]. But Leeuwenkamp et al. showed that the bioavailability of orally administered strontium is only about 20% [18]. Therefore, in order to increase the local strontium dosage new types of strontium enriched calcium phosphate bone cements have recently been developed to achieve a local release of strontium ions into the bone defect [19].

In this paper we report on the influence of these strontium enriched biomaterials on the behavior of osteoblast-like cells and the formation of their mineralized extracellular matrix (mECM) as analyzed by time-of-flight secondary ion mass spectrometry (ToF-SIMS). ToF-SIMS is a highly surface-sensitive and chemically specific analytical technique for both inorganic and organic matter. A focused primary ion beam is used to generate ionized molecular fragments from a solid sample in ultra-high vacuum. These secondary ions are then separated by their mass-to-charge ratio using a time-of-flight mass analyzer [20, 21]. By carefully analyzing the mass spectrum the chemical composition of the sample can be reconstructed. As the primary ion beam can be rasterized across the sample surface an image of the intensity distribution of a selected mass signal can be obtained [22]. By using an additional ion gun (sputter gun), which is much more intensive than the analysis gun (primary ion gun), it is possible to remove a small area of the sample layer by layer to get three-dimensional mass information. This technique has been applied to a wide range of materials. The development of polyatomic ion sources which cause less residual damage of the ion-impacted surface in recent years made it possible to investigate also biological samples [23, 24]. Major improvements were seen during the last two decades, and currently the application of ToF-SIMS to the analysis of tissue sections and single cells is quickly advancing [25, 26]. Several groups have shown that ToF-SIMS is a powerful technique to characterize cells [27] and to achieve 3D analysis of cells [28–30]. It was also demonstrated that it is possible to investigate the mineralized extracellular matrix of osteoblasts [31].

Here we use ToF-SIMS to investigate whether strontium is incorporated in the mineralized extracellular matrix (mECM) and whether there is strontium uptake by osteogenically differentiated human mesenchymal stem cells (hMSCs). Therefore the distribution of the strontium mass signal in mineralized extracellular matrix of osteoblast-like cells cultured on two different strontium enriched bone cements was analyzed with high spatial resolution. Also the strontium intensity in the osteoblast-like cells relocated from the biomaterials to silicon wafers was monitored, which may help to understand the beneficial effect of strontium on osteoblasts better.

## Methods

### Cement modification with strontium (II)

Strontium (II) was introduced into a hydroxyapatite forming, α-tricalcium phosphate based cement by (a) the addition of strontium carbonate or by (b) complete substitution of CaCO_3_ (a component of the standard cement precursor formulation) by SrCO_3_ as described in detail elsewhere [19]. Briefly, cement precursor powder (InnoTERE GmbH, Radebeul, Germany) was composed of α-tricalcium phosphate (α-TCP), dicalcium phosphate (monetite), calcium carbonate (CaCO_3_) and hydroxyapatite (HA). In substitution-type samples, CaCO_3_ was replaced with SrCO_3_ (samples denoted as S100), whilst 10 wt-% SrCO_3_ were added to the precursor powder in samples referred to as A10. Therefore, in S100 samples a homogenous substitution of Ca^2+^- by Sr^2+^- ions could be obtained, whereas A10 samples are characterised by SrCO_3_ clusters embedded in a Sr-free cement matrix [19]. Cylindrical samples of 10 mm diameter and approx. 1 mm height were manually prepared by moulding a paste prepared from the precursor powder mixed with 400 μL g^-1^ 4% Na_2_HPO_4_ (Sigma Aldrich, Taufkirchen, Germany) solution in water. Samples were cured for 4 days in water-saturated atmosphere at 37°C and subsequently sterilised (γ-radiation, 25 kGy). A Sr-free standard calcium phosphate cement (CPC) prepared according to the same protocol was used as a control material.

### Cell culture

Primary human mesenchymal stem cells (hMSC) isolated from the bone marrow of 3 donors kindly provided by the Medical Clinic I, Dresden University Hospital “Carl Gustav Carus” (Prof. Martin Bornhäuser and co-workers) were used after given consent. The ethics commission of Technische Universität Dresden approved application of hMSC for *in vitro* experiments. Cells were cultured in α-MEM containing 9% fetal calf serum (FCS), 10 U mL^-1^ penicillin, 100 μg mL^-1^ streptomycin and 1% L-glutamine (all purchased from Biochrom, Berlin, Germany) at 37°C and 5% CO_2_. Two different types of cell samples were prepared. On the one hand cells were seeded onto biomaterials (CPC, A10 and S100) to study matrix formation and mineralization *in vitro*. 2 · 10^4^ cells of the 5th passage were cultured for 21 days in the presence of osteogenic supplements (10^-8^ M dexamethasone, 5 mM β-glycerophosphate and 0.05 mM ascorbic acid 2-phosphate, all purchased from Sigma Aldrich, Taufkirchen, Germany). On the other hand relocated cells were used to study the strontium content inside the cells. Therefore cells cultured on cement samples were detached using trypsin/EDTA (Invitrogen) for 15 min, transferred to silicon wafers (Si-MAT, Kaufering, Germany) and allowed to adhere for 24 h (CPC r, B10 r, S100 r). Several control groups on silicon wafers were also prepared. 2 · 10^4^ cells of the 5th passage were seeded onto silicon wafers and cultured in differentiation medium for one (Si1a-c) or 21 days (Si21d). In case of some of the cells cultured on silicon wafers for 21 days, the medium was further supplemented with 0.1 mM (SrCl_2_ 0.1) and 1.0 mM SrCl_2_ (SrCl_2_ 1.0), respectively. The strontium chloride was purchased from Sigma Aldrich. One load of the cell samples cultured with 1.0 mM SrCl_2_ was relocated afterwards on a silicon wafer (SrCl_2_ 1.0 r). All cell samples were washed with phosphate buffered saline (PBS, Invitrogen) and fixed with 3.7% glutaraldehyde (Sigma Aldrich), dehydrated in ethanol (VWR, Darmstadt, Germany) and subsequently critical-point dried (CPD 030, Bal-Tec, Liechtenstein). Details of all samples are summarized in Tables 1 and 2.

**Table 1:** Samples used to study incorporation of strontium in the mECM

Sample name	Description	hMSCs cultured in osteogenic differentiation medium for
**CPC m**	Pure CaP-bone cement.	21 days
**A10 m**	CaP-bone cement with 10 wt-% SrCO_3_ added.	21 days
**S100 m**	CaP-bone cement with SrCO3 substituting CaCO_3_.	21 days

**Table 2:** Samples used to compare the strontium content inside the osteogenically differentiated hMSCs

Sample name	Description	hMSCs cultured in osteogenic differentiation medium for
**Si1d a**	No strontium addition.	1 day on a silicon wafer
**Si1d b**	No strontium addition.	1 day on a silicon wafer
**Si1d c**	No strontium addition.	1 day on a silicon wafer
**Si21d**	No strontium addition.	21 days on a silicon wafer
**CPC r**	Pure CaP-bone cement.	21 days on cement, relocated on a silicon wafer and cultured for one day.
**A10 r**	CaP-bone cement with 10 wt -% SrCO_3_ added.	21 days on cement, relocated on a silicon wafer and cultured for one day.
**100 r**	CaP-bone cement with SrCO_3_ substituting CaCO_3_.	21 days on cement, relocated on a silicon wafer and cultured for one day.
**SrCl**_**2**_**0.1**	0.1 mM SrCl_2_ solution added to the medium	21 days on a silicon wafer
**SrCl**_**2**_**1.0**	1.0 mM SrCl_2_ solution added to the medium	21 days on a silicon wafer
**SrCl**_**2**_**r**	1.0 mM SrCl_2_ solution added to the medium	21 days, relocated on a silicon wafer and cultured for one day.

### ToF-SIMS analysis

Pure cement samples and the critical-point dried cell samples were used for ToF-SIMS analysis. ToF-SIMS data were acquired using a TOF.SIMS 5 instrument (ION-TOF, Münster, Germany) equipped with a 25 keV bismuth primary ion-source and a 2 keV O_2_ sputter ion- gun. To investigate the pure cements and the mECM of the cement cultured cells, sample imaging was performed within an area of 120 × 120 μm^2^ to 250 × 250 μm^2^ using Bi_3_^+^ cluster ions. Each scan provides an image with 128 × 128 pixels and an approximate pixel size of 5–10 μm. The target current was 0.3 pA - 0.4 pA and the ion dose density was about 1∙10^12^ 1/cm^2^. Images were recorded using the high current bunch mode with high mass resolution (*m*/Δ*m* FWHM > 4000). For calibration the masses of the molecules CH_3_^+^, C_2_H_3_^+^, C_3_H_5_^+^ and C_7_H_7_^+^ were used. To compare the ToF-SIMS mass images with optical images the 2D mode of a PLu neox 3D optical profiler (Sensofar, Terrassa, Spain) equipped with a blue LED at 460 nm wavelength was used.

For 3D-analysis of the osteoblast-like cells, cells were relocated from the cements to silicon wafers to avoid mixing effects close to the cement/cell interface. For the 3D-analysis the oxygen sputter gun was used because further experiments have shown that strontium shows a higher ionization rate by using O_2_^+^ instead of C_60_^+^ or Cs^+^. The comparison between 3D profiles obtained by different analytical methods showed that virtually no differential sputtering occurred during the depth profiling using the oxygen sputter gun (Additional file 1). O_2_^+^_-_ sputtering was performed on a 400 × 400 μm^2^ area with a kinetic energy of 500 eV. The current at the target ranged between 90 nA - 100 nA. The analysis sequence included a sputter cycle of 1 s followed by acquisition of 10 scans in high current bunch mode in the respective crater center of 100 × 100 μm^2^. Data evaluation was performed using the software TOF-SIMS Surface Lab 6.3 (ION-TOF GmbH, Germany). For 3D reconstruction of the depth profiles the NESAC/BIO toolbox ZCorrectorGUI based on MATLAB was used [32].

In order to compare the relative intensities of the strontium signal from the depth profiles of different samples the following procedure was carried out:

1. Three adherent cells of each sample were depth profiled using the same raster size, primary ion dose per cycle (1∙10^14^ 1/cm^2^), and sputter dose per cycle (1∙10^16^ 1/cm^2^).
2. For evaluating the data the same area size of region of interest (ROI) was chosen for every cell and the data were reconstructed from the ROI. The strontium signal was summed up over 100 sputter cycles from each profile and normalized to total counts.

The cell size was determined using the 3D mode of the PLu neox 3D optical profiler (Sensofar, Terrassa/Spain). The thickness of the cells varies between 250 – 400 nm. This results in estimated sputter rates of 0.2 – 0.3 nm/s.

## Results and discussion

**Figure 1:**
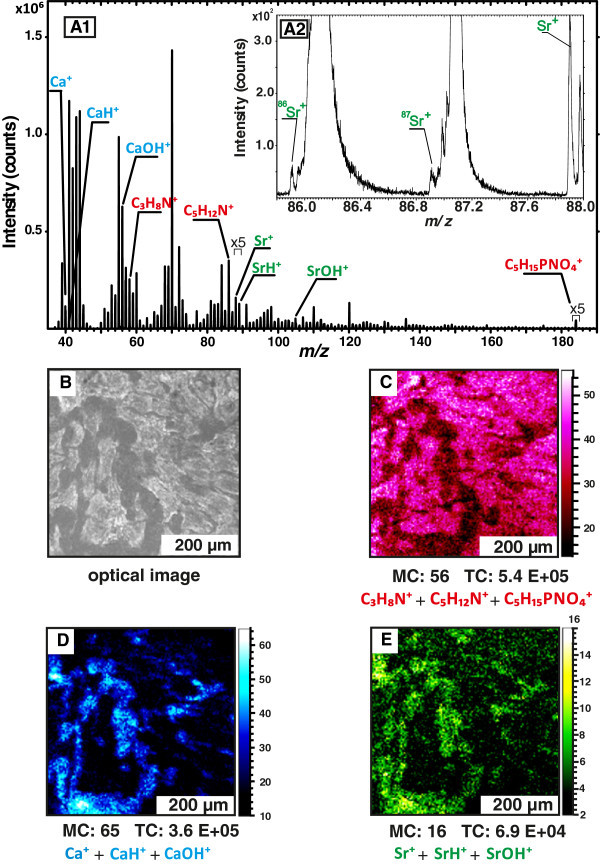
**Mass spectra (A1 and A2), optical image (B) and SIMS mass images (C-E) of strontium substituted calcium phosphate cement cultured with hMSCs in osteogenic differentiation medium for one day (S100 1d).** Figure **A1** shows the labeled masses which were used to generated the SIMS mass images. A typical mass pattern of the strontium isotopes is shown in Figure **A2**. The spatial distribution of the cell mass signals **(C)**, calcium signals **(D)** and strontium signals **(E)** are shown in the SIMS mass images. The spatial patterns of mass signals offer the same morphological information as the microscope image **(B)**, in which the cells are shown in white and the S100 cement in grey.

A typical mass spectrum of an area on the strontium substituted calcium phosphate cement cultured with hMSCs in osteogenic differentiation medium for one day (S100 1d) is shown in Figure 1 A1. In previous studies characteristic mass signals of ToF-SIMS spectra originating from the cell membrane have been attributed unequivocally to characteristic biomolecules from the cells [33]. These characteristic masses include *m/z* = 184 u, being phosphatidylcholine, which is part (head group) of the most abundant class of phospholipids in mammalian cells. The *m/z* = 184 u ion (C_5_H_15_PNO_4_^+^) has a characteristic fragmentation pattern in parallel, and the major fragments are ions at *m/z* = 86 u (C_5_H_12_N^+^) and *m/z* = 58 u (C_3_H_8_N^+^). These can clearly be seen in the mass spectrum in Figure 1 A1. The phosphatidylcholine signal and the signals of its fragments are normally homogenously distributed across the cell surface. Figure 1C shows the distribution of the ions *m/z* = 184 u, 86 u and 58 u on a sample area which represents the same spot as in the microscope image in Figure 1B. In the microscope image the cells are shown in white and the uncovered cement in grey. In the SIMS mass images the intensity of the pixels correlate with the intensity of the mass signal. A bright pixel indicates high intensity of the signal; a dark pixel indicates low intensity. The mass image of the cell signals matches the microscope image. Thus it is possible to evaluate space-resolved information about the sample given by the distribution of the mass signals. As the biomaterial contains calcium and strontium we find these cations and related species in the spectrum (Figure 1 A1). Figure 1 A2 shows the typical pattern of the isotope distribution of strontium, which supports the assumption that the mass signal *m/z* = 87.9 u represents strontium. In Figure 1D the distribution of calcium ions is shown. The calcium image is a negative to the cell signal image, as we only measured the first monolayers of the sample surface (static mode). The distribution of the strontium signals, shown in Figure 1E, is equal to the distribution of the calcium signals (Figure 1D), but the intensity is much lower, as the concentration of strontium in the S100 biomaterial is less than of the calcium.

### Investigation of the mECM on different bone cements

**Figure 2:**
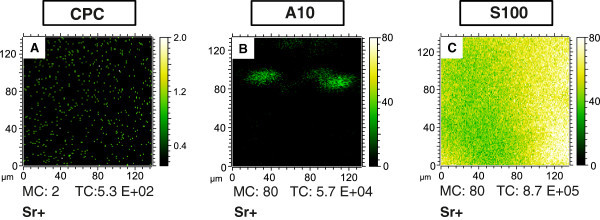
**Strontium distribution on the surface of three biomaterials CPC (A), A10 (B) and S100 (C).** For **B** and **C** the same intensity scale was used.

**Figure 3:**
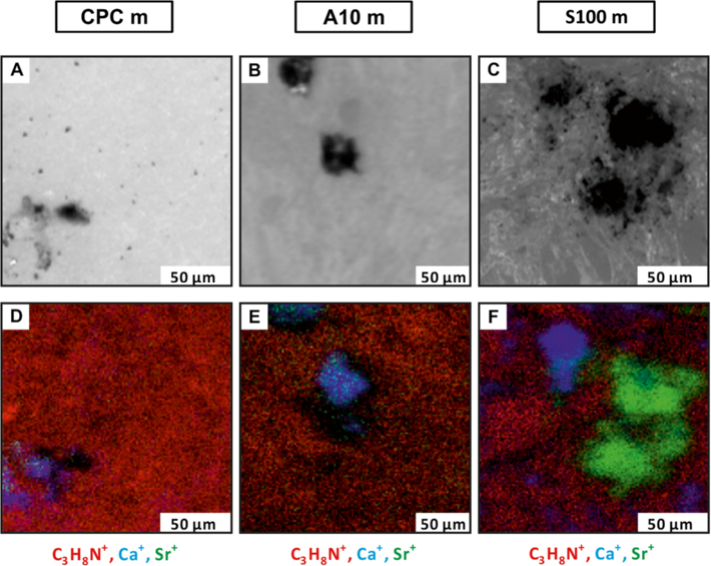
**Light optical microscope images (A-C) and SIMS mass images (D-E) of the mECM synthesized by osteogenically differentiated hMSCs cultured for 21 days on three different bone cements (CPC m, A10 m, S100 m).** In the microscope images the cells are shown in white, the uncovered cements in grey and the mECM in black **(A-C)**. In the SIMS images the cell mass signal (C_3_H_8_N)^+^ is shown in red, the calcium signal in blue and the strontium signal in green **(D-F)**.

In the following, two different strontium enriched bone cements are compared with pure calcium phosphate cement (CPC) as control. The strontium enriched bone cements S100 and A10 differ in the distribution of strontium (II) on the cement surfaces as can be seen in Figure 2. A homogenous substitution of Ca^2+^- by Sr^2+^- ions could be obtained in the case of the new biomaterial S100 (Figure 2C). The new bone cement A10 is characterized by SrCO_3_ clusters (size about 30 μm), embedded in a Sr-free cement matrix (Figure 2B). The differences of the two Sr-enriched biomaterials result from the two different preparation methods [19]. Virtually no strontium was detected on the surface of the pure CPC (Figure 2A). Osteogenically differentiated hMSCs were cultured on the three different bone cements. To compare the chemical information of the mineralized extracellular matrix (mECM), synthesized by the osteoblast-like cells, the same spot of the sample was imaged using the microscope and the ToF-SIMS. Representative pairs of images for every material are shown in Figure 3. The first row of Figure 3 (A-C) shows the microscope images, and in the second row (Figure 3D-F) the corresponding ToF-SIMS overlays of mass images are depicted. As before, in the microscope images (Figure 3A-C) the cells are shown in white, the uncovered cement in grey and the mineralized extracellular matrix (mECM) is shown in black. In the ToF-SIMS overlays the cell mass signal C_3_H_8_N^+^ is shown in red, the calcium signal in blue and the strontium signal in green. The cell mass signals in the SIMS mass images exhibit the same shape as the white spots in the microscope images, which represent the cells. The calcium signal is found in the regions of the uncovered cement surface and the mECM. In comparison to the control group (CPC, Figure 3D) a very high strontium signal is observed in the region of the mECM in the ToF-SIMS image of the S100 sample (Figure 3F). Furthermore, only a weak Ca signal is detected in the mECM due to the low calcium concentration. But it is found that the intensities of calcium and strontium are not homogenously distributed over the different spots of the mECM. Until know we have no explanation for this finding. In the case of the pure CPC and A10 a high intensity of the Ca-signal was observed in the region of the mECM. Here strontium was only found in a few spots of the mECM, and if so, only a very low intensity of strontium was detected (Figure 3D-E). In the case of the strontium enriched biomaterials previous study showed that strontium is released from the biomaterials into the medium [19]. This indicates that the cells used most likely the free Sr^2+^-ions in the medium to build up their mECM. Since the A10 samples are characterized by an inhomogeneous distribution of strontium the cells are exposed to different strontium concentrations. Therefore strontium could be found in only a small number of matrix nodules.

Previous studies have shown that strontium is incorporated in the matrix nodules after cells were cultured with strontium salts [11]. How the strontium uptake in the mECM takes place is still unknown. It seems likely that the strontium ion reacts like the calcium ion. Various mechanisms how calcium is taken up into the mineralized extracellular matrix have been postulated: i) a cell-independent process where collagen associates with noncollagenous proteins to produce native fibrils, which mediate mineral nucleation [34]; ii) a cell-controlled mechanism by which Ca^2+^ and phosphate ions are accumulated in matrix-vesicles extracellulary or iii) calcium and amorphous calcium phosphate are stored inside the cells and transported via vesicles to the ECM [35]. The same mechanisms may be responsible for the incorporation of strontium into the mECM. As there is no native storage of strontium inside the cell, it is more likely that strontium is incorporated extracellulary into the mECM due to a Ca/Sr exchange.

### Monitoring the strontium signal inside the cells

In a next step we compared the strontium content inside the osteogenically differentiated hMSCs cultured on the strontium enriched bone cements with cells cultured on the pure calcium phosphate cement (CPC) and the silicon wafer (Si21d). To analyze the strontium distribution inside the cells a sputter gun was used to remove the cell layer by layer. Between the sputter cycles the exposed surfaces were analyzed using the primary ion gun. Since cells are not planar and as the sputter gun was arranged at a 45° angle to the sample various removal rates were obtained from different spots on the sample. Therefore the thinner parts of the cell are earlier removed completely than the thicker parts.

**Figure 4:**
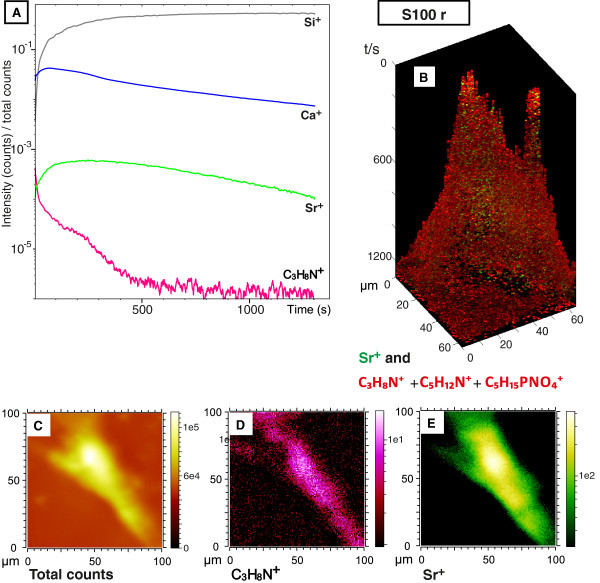
**3D analysis of an osteogenically differentiated hMSC.(A)** depth profile of an osteogenically differentiated hMSC cultured on S100 relocated after 21 days on a silicon wafer(S100 r). The signal intensity is shown over the whole analysis area. The estimated thickness of the cell is 280 nm. **(B)** 3D reconstruction of the depth profile showing the distribution of strontium and the cell signals C_3_H_8_N^+^, C_5_H_12_N^+^ and C_5_H_15_PNO_4_^+^. The value of the z axis has no physical meaning and depends on the number of layers in the 3D profile. The SIMS mass images show the lateral distribution summed over the whole depth profile of the total ions **(C)**, cell mass signal C_3_H_8_N^+^**(D)** and the strontium signal **(E)**.

**Figure 5:**
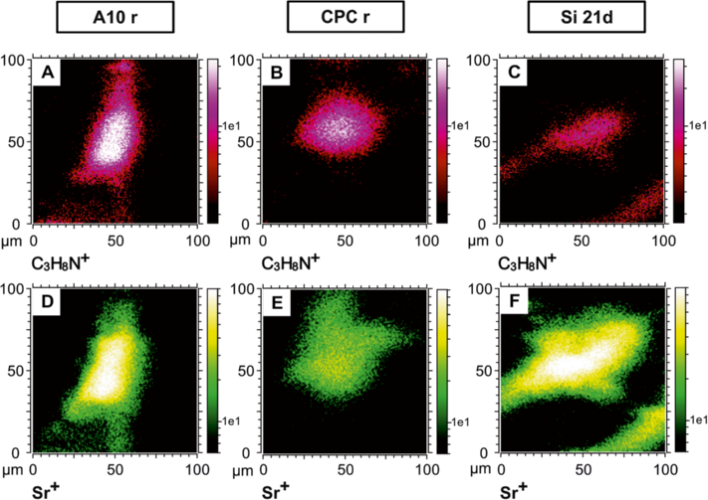
**SIMS mass images of the cell signals (A-C) and strontium signals (D-F) of osteoblast-like cells.** Cells were cultured in osteogenic differentiation medium on A10 and on the control groups CPC cement and silicon wafer (Si 21d). Cells cultured on the cements were relocated on a silicon wafer afterwards (A10 r, CPC r). For each mass signal of the different samples the same intensity scale was used.

A depth profile of an osteoblast-like cell cultured on S100 cement for 21 days and relocated afterwards by trypsination and subsequent re-seeding on a silicon wafer (S100 r) can be seen in Figure 4A. The intensities of the signals are shown for the cell thickness of 280 nm and summed up over the analyzed area which can be seen in Figure 4C-E. As the cells were relocated on silicon wafer the Si-signal increases with sputter time and remains nearly constant after 300 s of the depth profile measurement. During the first seconds of sputtering a decrease of the cell signal was observed, which indicates that the phospholipid membrane had been removed. After entering the interior of the cell an increase of both the strontium and the calcium signal were found. Both signals remain fairly constant until the silicon wafer is reached, then the signals of Sr^+^ and Ca^+^ decrease again. It is known that oxygen sputtering can cause sample degradation which leads to fragmentation of the organic compounds during depth profiling. This can also explain the loss of the lipid signal in the depth profile in Figure 4A. In the mass spectrum of the last 100 s of the depth profile the signal of C_3_H_8_N^+^ and also other organic compounds like C_3_H_3_O^+^ are still observed (Additional file 2). Therefore the lateral distribution of the cell signal C_3_H_8_N^+^ during the depth profile is shown in Figure 4D. For better comparison the total ion image is shown in Figure 4C. The distribution of the strontium signal can be seen in Figure 4E. In these SIMS mass images the intensities obtained at different z-values are summed up, i.e. the images represent depth integrals. The strontium signal is found in the same region as the cell signal. As strontium could be detected over the whole depth profile and could not be detected in the outer part of the cell this indicates that strontium is uniformly distributed inside the cell. To confirm the assumption that strontium has indeed moved into the cell a 3D reconstruction including a z-correction [32] from the SIMS data of the depth profile was made. The distribution of the strontium signal (green) and the summed cell signals of C_3_H_8_N^+^, C_5_H_12_N^+^ and C_5_H_15_PNO_4_^+^ (red) are shown in Figure 4B. Due to the O_2_^+^ - sputtering the cell signals are unfortunately too weak to reconstruct the whole cell membrane. But we collected still enough signal intensity to estimate the shape of the cell. From this 3D illustration it can also be concluded that the strontium signal is only distributed inside the cell. For further data evaluation only the 2D images were used since the same interpretation can be achieved with a better validity. In Figure 5D the strontium distribution inside a cell cultured on the strontium enriched calcium phosphate cement A10 can be seen. The same distribution of strontium in the cell could be observed. This indicates that strontium released by the cements can enter the cells.

**Figure 6:**
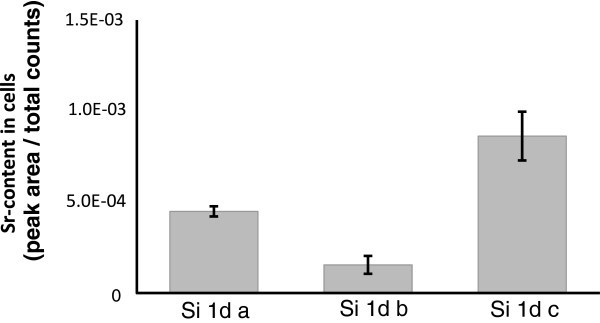
**Strontium content in the hMSCs cultured on silicon wafers for one day from three different cell donors (Si 1d a, b, c).** Data are expressed as the mean ± standard deviation (n = 3).

**Figure 7:**
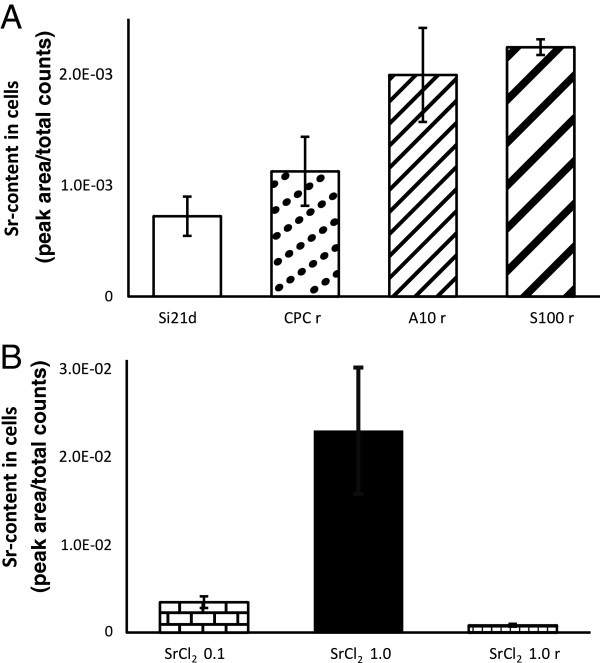
**Sr content in osteogenically differentiated hMSCs.(A)** Strontium content in osteogenically differentiatedhMSCs from the same donor as in Figures 4 and 5 cultured onthe three different bone cements (CPC r, B10 r, S100 r) and ona silicon wafer for 21 days (Si 21d). **(B)** Strontium content in osteogenically differentiated hMSCs cultured on silicon wafers for 21 days with SrCl_2_ in the growth medium. The first and second column show the Sr content in cells cultured with 0.1 mM and 1.0 mM SrCl_2_, respectively in the medium. The third column shows the Sr content in cells, which were relocated after 21 days with 1.0 mM SrCl_2_ in the medium (SrCl_2_ r). Data are expressed as the mean ± standard deviation (n = 3).

As can be seen in Figure 5, strontium was also detected inside the cells of the control groups, relocated cells from the pure CPC (CPC r, Figure 5E) and within cells cultured on the silicon wafer (Si21d, Figure 5F). No strontium was added to the osteogenic differentiation medium of all samples and no Sr could be detected by AAS (atomic absorption spectroscopy) in the purchased and standardized medium (data not shown). But strontium is a trace element and appears often with calcium. It also occurs in enzymes, which might be the reason why strontium was found in the negative control groups as well (Figure 5E-F). The strontium content in hMSCs of three different patients cultured for one day was analyzed. Therefore three cells of each donor were depth profiled. For data evaluation the same area size of region of interest was used. In Figure 6 it can be seen that the intensity of the strontium signals differs, so it can be suggested that the strontium content inside the cells depends on the cell donor. The cells in Figures 5 and 7 belong to the same donor. The cells investigated in Figure 6 were chosen from random donors to demonstrate the high variance between cells from different donors.

After 21 days of cultivation the strontium concentration inside the cells might be lower as in the cells which were only cultured for one day due to the proliferation. In Figure 5F the strontium content in the cells cultured on a silicon wafer for 21 days is almost as high as in the cells, cultivated on A10 (5D). This leads to the assumption that strontium is accumulated in the cells. However, to compare the strontium content in the cells cultured on the different biomaterials and substrates the hMSCs from the same donor were used. Again three cells on each type of substrate were measured. The strontium peak areas of the mass spectra of the different samples are compared in Figure 7A. The highest strontium content was found in the osteoblast-like cells cultured on the strontium enriched cements (S100 r and A10 r), and the lowest was found in the cells cultured for 21 days on a silicon-wafer (Si21d). A high variation in the strontium content of the A10 cells could be observed. As only three cells were measured the inhomogeneity of the A10 cement sample could cause such variation. In the case of the biomaterials the cells were relocated after 21 days on silicon wafers. Only perfectly adherent cells were chosen for depth profiling but as the relocated cells were no longer exposed to additional strontium, and the relocation causes stress for the cells the real strontium content could be different afterwards. To investigate the influence of the relocation, osteoblast-like cells were cultured with 1.0 mM SrCl_2_ for 21 days and one load was relocated afterwards by trypsination and subsequent re-seeding on a silicon wafer (SrCl_2_ 1.0 r). To clarify whether the intensity of the strontium signal as obtained with ToF-SIMS can be used as a measure of the strontium concentration; cells were cultured on silicon wafers with two different SrCl_2_ concentrations. The strontium content inside the cells of these control groups can be seen in Figure 7B. There is a large difference between the strontium intensity of the cells cultured with 0.1 mM and 1.0 mM SrCl_2_. This diagram indicates that it is possible to detect the comparative difference of strontium contents inside the cells, which were exposed to different strontium concentrations via ToF-SIMS. Furthermore it proves that the strontium content in cells will be higher if the cells are exposed to a higher concentration of strontium. The strontium content in the relocated cells (SrCl_2_ 1.0 r) is much lower than in the original sample (SrCl_2_ 1.0). The relocation of the cells influences the strontium signal. That means that the original strontium content of the cells cultured on the cements could be much higher than it is observed in Figure 7A.

However, strontium could be found in the mineralized extracellular matrix (mECM) synthesized by the osteoblast-like cells cultured on the cements and strontium could be detected in the osteoblast-like cells of every sample type. But a clear difference in the amount of strontium could be observed. The higher the strontium concentration in the medium the higher the intensity of the strontium signals in the osteoblast-like cells and in the mECM. The mechanism of the strontium incorporation into the mECM cannot be clarified from this study. Therefore further biomolecular investigations are necessary. As mentioned before strontium enhances the replication and differentiation of pre-osteoblastic cells and the activity of functional osteoblasts. Due to that the rate of bone matrix synthesis increases [9]. The molecular targets of strontium are still being investigated. As strontium is a divalent ion and chemically close to Ca^2+^, Sr^2+^ may act on similar cellular targets as the calcium ion [36]. In the case of calcium the uptake into osteoblasts takes place due to different families of calcium ion channels inside the phospholipid membrane [37]. As the ionic radius of strontium is twice that of calcium, it is more likely that strontium passes the membrane using one of the non-selective ion channels. It was demonstrated that strontium plays an important role in signaling pathways in osteoblasts. On the one hand it activates the calcium sensor receptor (CaSR) [38] on the other hand strontium is involved in the Wnt signaling pathway. These combined interactions of Sr^2+^ result in increased osteoblastic cell replication, osteoblast gene expression and cell survival [36]. These beneficial effects on the osteoblasts depend on the concentration of the strontium ions inside the cells. A high strontium concentration leads to a high impact on the signaling pathways. As the strontium content inside the cells cultured on the strontium enriched biomaterials was higher than in the control groups the newly developed calcium phosphate cements S100 and A10 are promising implant materials for the use in systemically altered bone.

## Conclusion

It was previously shown that newly developed strontium enriched bone cements release strontium to the medium [19]. Here we could demonstrate that the released strontium is incorporated into the mineralized extracellular matrix (mECM) as well as enriched inside the osteogenically differentiated hMSCs. In comparison to the control group we detected a definitely higher amount of strontium in the mECM of the osteoblast-like cells cultured on the strontium substituted bone cement S100. The present data are in accordance with previous studies showing that strontium is incorporated in the mECM [11] and in newly formed human bone [39]. Our results prove that strontium ions from artificial biomaterials indeed pass the cellular membrane and accumulate inside the osteoblast-like cells. The strontium was found to be uniformly distributed in the interior of the cells. The strontium content inside the cells cultured on the strontium enriched bone cements is much higher than in cells cultured on the control groups. As strontium is known for its beneficial effect on osteoblasts the strontium release is a promising property of the strontium enriched calcium phosphate cements for their use as an implant material for osteoporotic bone. In contrast to the A10 samples a homogenous distribution of Sr^2+^-ions could be obtained in the S100 samples. Therefore the osteoblast-like cells cultured on the S100 biomaterial are exposed to the same Sr concentration. This results in a higher strontium concentration in the osteoblast-like cells, cultured on the S100 biomaterial and in their mECM. In conclusion, the strontium substituted calcium phosphate cement S100 could be proposed as a promising biomaterial for the treatment of bone defects in osteoporotic patients.

Additional file 1: **A) 3D profile of a cell cultured on S100 for 21 days and relocated to a silicon wafer obtained by the PLu neox 3D optical profiler before the cell was depth profiled with ToF-SIMS.** B) 3D reconstruction of the same cell as in A) using SIMS data. C) 2D image in false color map of the same cell. The black line indicates the position of the corresponding z profile in D). To compare the 3D profiles obtained with different analytical methods we have to take into account that we cannot proper scale the z axis of the SIMS depth profile with the applied software tool and it is difficult to look at the exact same cross-section of the cell. Considering these facts the 3d profiles look nearly the same. This leads us to the assumption that rarely differential sputtering occurs. (PDF 305 KB)

Additional file 2: **Mass spectra of the last 100 s of a depth profile from a cell cultured on S100 for 21 days and relocated to a silicon wafer.** Organic compounds like the C_3_H_3_O^+^ ion, which is most likely a fragment of an amino acid can still be observed. (PDF 383 KB)
