# Supplementary material for: ToF-SIMS analysis of osteoblast-like cells and their mineralized extracellular matrix on strontium enriched bone cements
Source: Biointerphases. 2013 Jul 23;8(1):17. doi: 10.1186/1559-4106-8-17 (PMC5849209; doi:10.1186/1559-4106-8-17)

Additional File 2: A) 3D profile of a cell cultured on S100 for 21days and relocated to a silicon wafer obtained by the PLu neox 3D optical profiler before the cell was depth profiled with ToF-SIMS. B) 3D reconstruction of the same cell as in A) using SIMS data. C) 2D image in false color map of the same cell. The black line indicates the position of the corresponding z profile in D). To compare the 3D profiles obtained with different analytical methods we have to take into account that we cannot proper scale the z axis of the SIMS depth profile with the applied software tool and it is difficult to look at the exact same cross-section of the cell. Considering these facts the 3d profiles look nearly the same. This leads us to the assumption that almost no differential sputtering occurs.

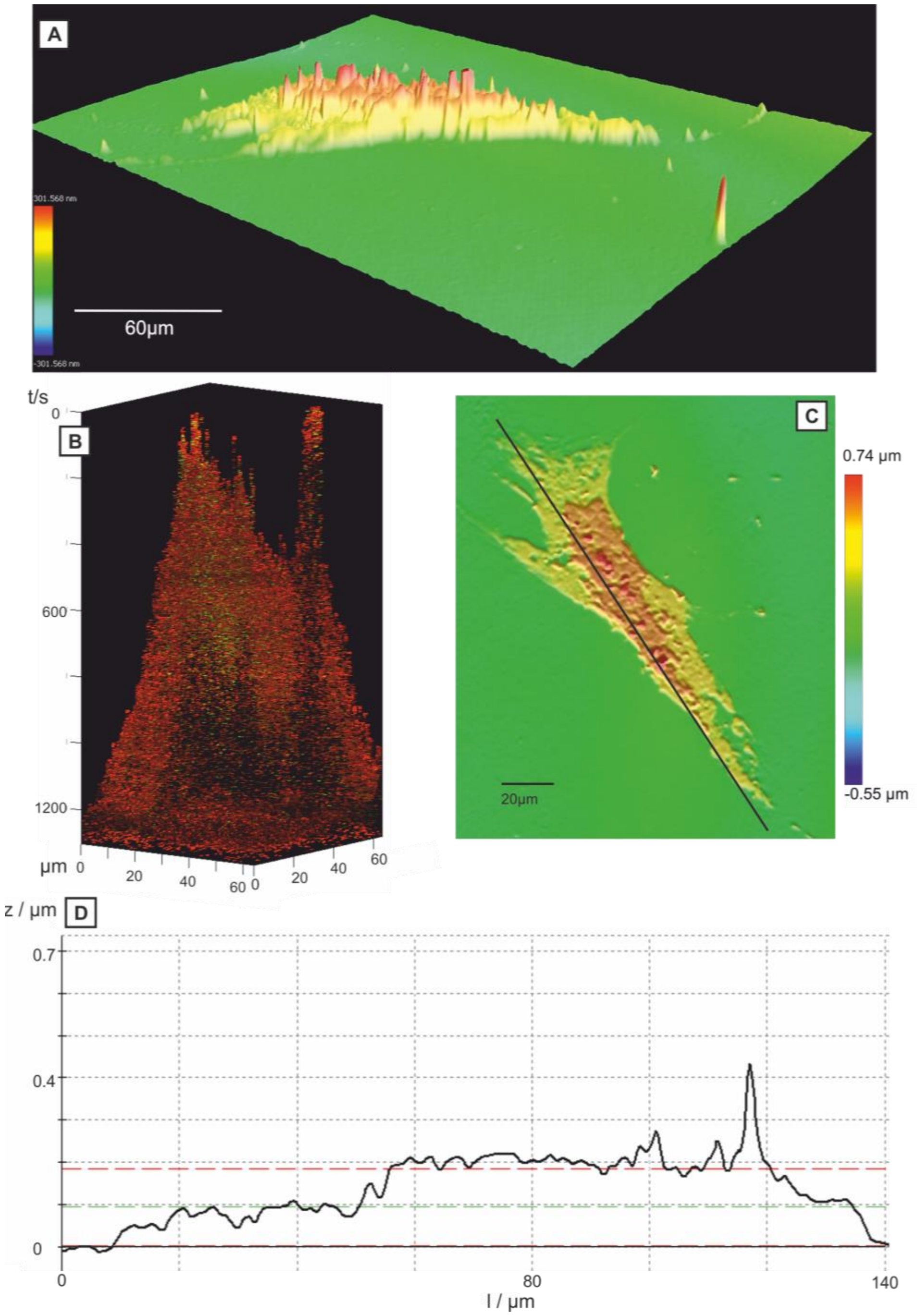

Supplement: Supplementary file 1 — Additional file 1: A) 3D profile of a cell cultured on S100 for 21 days and relocated to a silicon wafer obtained by the PLu neox 3D optical profiler before the cell was depth profiled with ToF-SIMS. B) 3D reconstruction of the same cell as in A) using SIMS data. C) 2D image in false color map of the same cell. The black line indicates the position of the corresponding z profile in D). To compare the 3D profiles obtained with different analytical methods we have to take into account that we cannot proper scale the z axis of the SIMS depth profile with the applied software tool and it is difficult to look at the exact same cross-section of the cell. Considering these facts the 3d profiles look nearly the same. This leads us to the assumption that rarely differential sputtering occurs. (PDF 305 KB) [file 13758_2013_17_MOESM1_ESM.pdf]
