# Supplementary material for: ToF-SIMS analysis of osteoblast-like cells and their mineralized extracellular matrix on strontium enriched bone cements
Source: Biointerphases. 2013 Jul 23;8(1):17. doi: 10.1186/1559-4106-8-17 (PMC5849209; doi:10.1186/1559-4106-8-17)

Additional File 1: Mass spectra of the last 100 s of the depth profile from a cell cultured on S100 for 21days and relocated to a silicon wafer. Organic compounds like the $C_3H_3O^+$ ion (Fig. B), which is most likely a fragment of an amino acid can still be observed. Of course the intensities of the lipid fragments like the $C_3H_8N^+$ ion (Fig. A) are much reduced since the cell membrane is almost gone at the end of the profile.

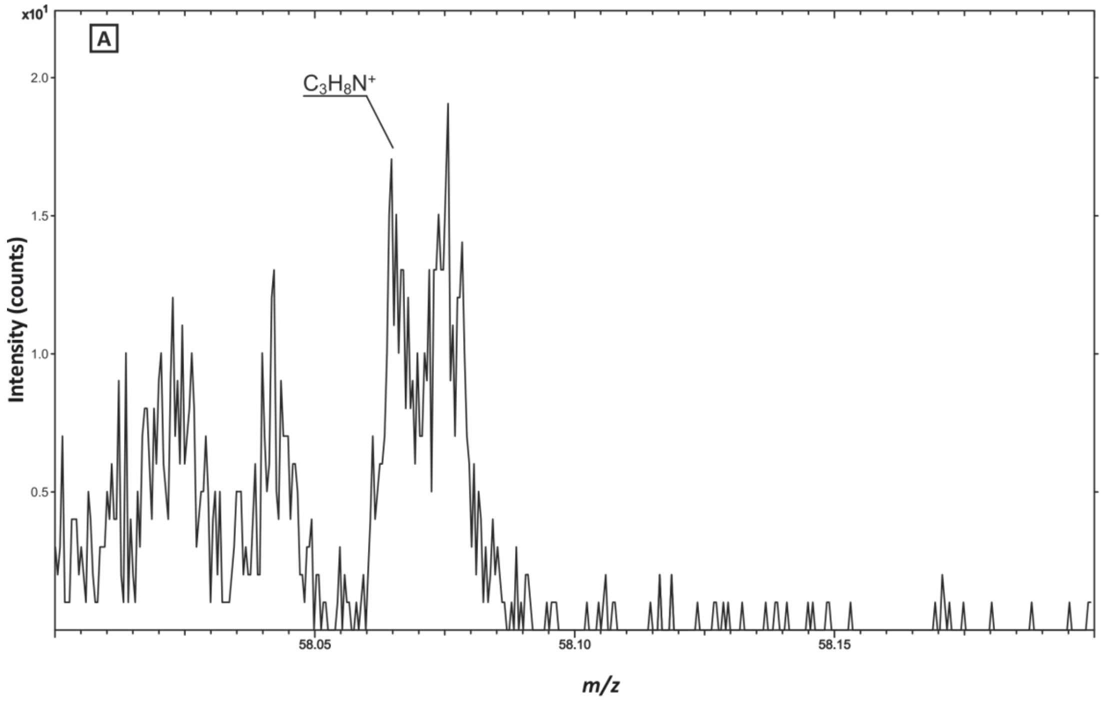

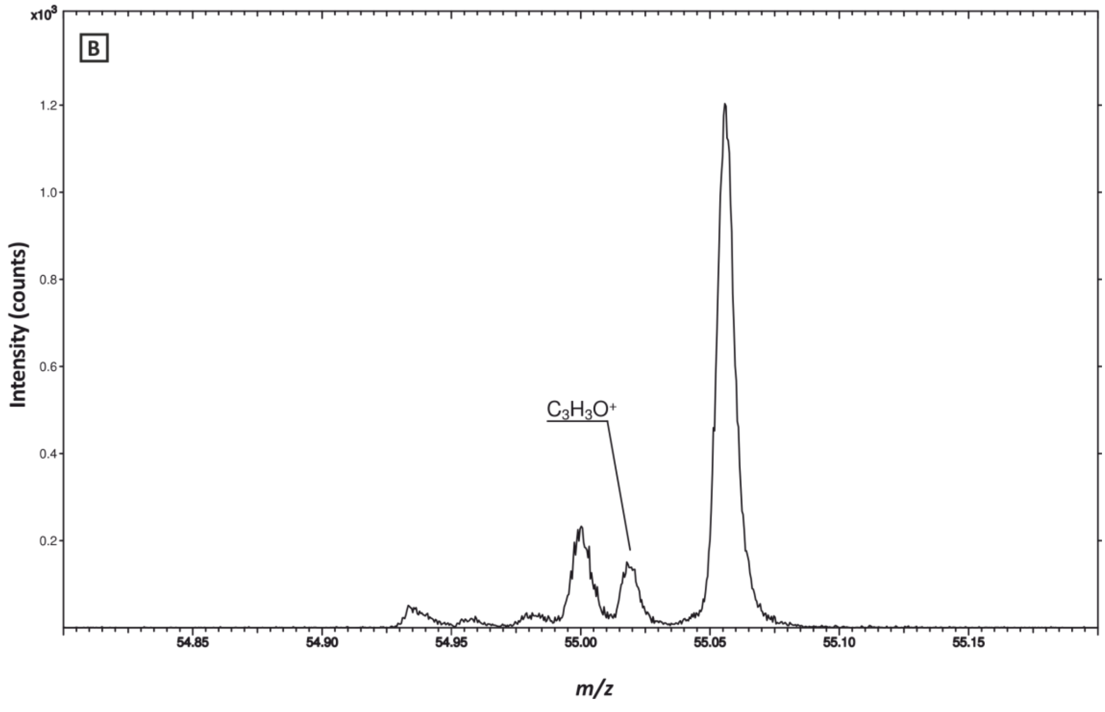

Supplement: Supplementary file 2 — Additional file 2: Mass spectra of the last 100 s of a depth profile from a cell cultured on S100 for 21 days and relocated to a silicon wafer. Organic compounds like the C3H3O+ ion, which is most likely a fragment of an amino acid can still be observed. (PDF 383 KB) [file 13758_2013_17_MOESM2_ESM.pdf]
